# Conditional Expression of *Wnt4* during Chondrogenesis Leads to Dwarfism in Mice

**DOI:** 10.1371/journal.pone.0000450

**Published:** 2007-05-16

**Authors:** Hu-Hui Lee, Richard R. Behringer

**Affiliations:** Department of Molecular Genetics, University of Texas M. D. Anderson Cancer Center, Houston, Texas, United States of America; Baylor College of Medicine, United States of America

## Abstract

*Wnts* are expressed in the forming long bones, suggesting roles in skeletogenesis. To examine the action of *Wnts* in skeleton formation, we developed a genetic system to conditionally express *Wnt4* in chondrogenic tissues of the mouse. A mouse *Wnt4* cDNA was introduced into the ubiquitously expressed *Rosa26 (R26)* locus by gene targeting in embryonic stem (ES) cells. The expression of *Wnt4* from the *R26* locus was blocked by a neomycin selection cassette flanked by *lox*P sites (floxneo) that was positioned between the *Rosa26* promoter and the *Wnt4* cDNA, creating the allele designated *R26^floxneoWnt4^*. *Wnt4* expression was activated during chondrogenesis using *Col2a1-Cre* transgenic mice that express Cre recombinase in differentiating chondrocytes. *R26^floxneoWnt4^; Col2a1-Cre* double heterozygous mice exhibited a growth deficiency, beginning approximately 7 to 10 days after birth, that resulted in dwarfism. In addition, they also had craniofacial abnormalities, and delayed ossification of the lumbar vertebrae and pelvic bones. Histological analysis revealed a disruption in the organization of the growth plates and a delay in the onset of the primary and secondary ossification centers. Molecular studies showed that *Wnt4* overexpression caused decreased proliferation and altered maturation of chondrocytes. In addition, *R26^floxneoWnt4^; Col2a1-Cre* mice had decreased expression of vascular endothelial growth factor (VEGF). These studies demonstrate that *Wnt4* overexpression leads to dwarfism in mice. The data indicate that *Wnt4* levels must be regulated in chondrocytes for normal growth plate development and skeletogenesis. Decreased VEGF expression suggests that defects in vascularization may contribute to the dwarf phenotype.

## Introduction

Wnt signaling has been implicated in the regulation of early patterning and initial outgrowth of the vertebrate limb bud [1]–[4]. More recently, several *Wnts* have been shown to be expressed in the developing long bones, suggesting that they may have roles in endochondral bone formation. In the developing chick skeleton, *Wnt4* and *Wnt9a* (previously known as *Wnt14*) are expressed in joint-forming regions, *Wnt5a* and *Wnt11* in the perichondrium, and *Wnt5b* in prehypertrophic chondrocytes of the growth plate [5]–[8]. Misexpression studies in chick embryos suggested that both *Wnt4* and *Wnt5a* can alter chondrogenesis and shorten limb growth, apparently by different mechanisms. *Wnt4* accelerates chondrocyte differentiation, whereas *Wnt5a* inhibits this process [5]. *Wnt9a* misexpression has been shown to induce the initiation of joint formation [6]. However, *Wnt9a* knockout mice formed joints but had ectopic cartilaginous nodules that was enhanced by loss of *Wnt4* [9]. *Wnt4*/*Wnt9a* double mutants also had some limb bone fusions apparently because of an inability to maintain joint cell identity [10]. Misexpression of *Wnt5b* as well as *Wnt5a* inhibits chondrogenesis in mice, but they appear to act differently. *Wnt5a* inhibits the transition from resting to proliferating chondrocytes in the growth plate, whereas *Wnt5b* promotes this transition as well as chondrocyte proliferation [11].

Wnt signaling components have also been investigated for their roles in skeletogenesis. Frb1, a secreted form of Frizzled that is a Wnt receptor, can function as an antagonist when misexpressed in long bone, causing shortening of skeletal elements, joint fusion, and delayed chondrocyte maturation [12]. In addition, constitutive expression of *Lef1* in chondrocytes stimulated chondrocyte maturation as well as replacement of cartilage by bone [13]. Furthermore, mice with a disruption of the *LDL receptor-related protein 5* (*Lrp5*) gene that encodes a Wnt co-receptor, showed decreased osteoblast proliferation [14]. In addition, *Lrp5*-deficient mice also displayed persistent eye vascularization. These bone and eye phenotypes are similar to the abnormalities associated with osteoporosis-pseudoglioma syndrome in human, caused by mutation of *LRP5* [15].

Most studies of Wnt signaling in skeleton development have been restricted to the chick model. However, the expression of *Wnts* appears to vary in different animal models. For example, in addition to the perichondrium of chick, *Wnt5a* expression was also found at the junction of proliferating and prehypertrophic chondrocytes in the radius and ulna of mice [11].

*Wnt4* expression has also been analyzed during kidney and female reproductive system development. *Wnt4* homozygous mutant mice died after birth due to a failure of pretubular cell aggregation, an essential step in the formation of nephrons of the kidney [16]. In addition, *Wnt4* mutant mice with an XX karyotype lacked female-specific genital ducts and developed male-specific genital ducts [17]. During chick skeletogenesis, *Wnt4* is initially expressed in joint-forming regions, and then is detected in the region of the joint capsule and surface articular chondrocytes [5], [18]. However at later stages, *Wnt4* expression in long bones is also detected in hypertrophic chondrocytes [18]. In the mouse, *Wnt4* is also expressed in forming joints and mesenchyme that will form the joint capsule [19]. The patterns of *Wnt4* expression in chick and mouse suggest roles in joint development and chondrocyte hypertrophy. In addition, the restricted pattern of *Wnt4* expression in bone-forming tissues suggests that its expression must be precisely controlled to coordinate normal bone and skeleton formation.

To study the actions of *Wnt4* during skeleton development, we created a conditional genetic system to express *Wnt4* during chondrogenesis. To accomplish this, we exploited the ubiquitously expressed *Rosa26* locus. The *ROSA26* mouse mutant was originally produced by infection of embryonic stem (ES) cells with a *ROSAβ-geo* retrovirus [20]. *Rosa26* heterozygotes express β-galactosidase (β-gal) reporter activity ubiquitously that initiates during preimplantation development at the morula-blastocyst stage. Examination of serial sections through 9.5 days post-coitus (dpc) *Rosa26* heterozygotes demonstrated β-gal activity in all cells [21]. *Rosa26* homozygous mutants are viable although they are recovered at a lower than expected frequency [21]. The *Rosa26* locus has been used to ubiquitously or conditionally express various gene products in mice [22]–[26]. Therefore, we exploited the *Rosa26* locus to express *Wnt4* in a Cre-dependent manner. We placed a drug selection cassette flanked by *lox*P sites between the *Rosa26* promoter and a mouse *Wnt4* cDNA, blocking *Wnt4* expression at the endogenous *Rosa26* locus. Cre expression should delete the blocking drug selection cassette, leading to *Wnt4* expression.

To examine the action of *Wnt4* during endochondral bone formation, we used *Col2a1-Cre* transgenic mice that express Cre activity in cartilage-forming tissues [27], [28]. We found that *Wnt4* expression in chondrogenic tissues alters skeletogenesis, resulting in skull abnormalities and dwarfism. These studies indicate that alterations in *Wnt4* expression can cause severe skeletal pathologies.

## Results

### *Dwarfism in R26^floxneoWnt4^; Col2a1-Cre* mutant mice

**Figure 1:**
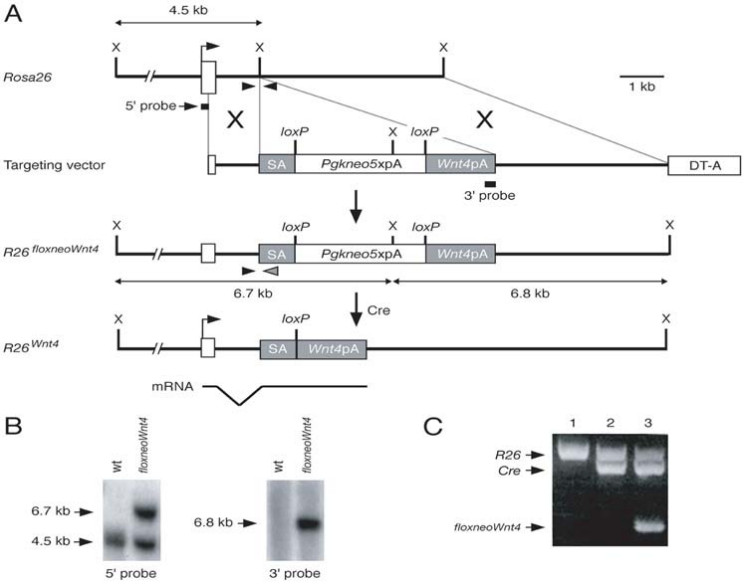
Generation of *R26^floxneoWnt4^* mice. A, Gene targeting strategy. Open box, *R26* exon 1; DT-A, diphtheria toxin expression cassette. SA, splice acceptor; X, *Xba*I. B, Southern analysis of ES cell clones. Genomic DNAs were digested with *Xba*I and detected by 5′ external or 3′ internal probes. C, PCR genotyping of *R26^floxneoWnt4^; Col2a1-Cre* mice. Primers shown in panel A (arrowheads) amplify *R26* wild-type (∼600-bp) and targeted (∼350-bp) alleles. *Cre*-specific primers yield a ∼550-bp band to identify mice carrying the *Col2a1-Cre* transgene.

A conditional genetic system was created to express *Wnt4* in a Cre-dependent manner, potentially in any tissue. We modified the ubiquitously-expressed *Rosa26* locus by gene targeting in ES cells (**Fig. 1A, B**). A mouse *Wnt4* cDNA was placed 3′ of a floxed neomycin resistance expression cassette, *floxneo*, which should block the transcription of the *Wnt4* cDNA from the *Rosa26* promoter. This block in transcription should be relieved by Cre recombinase-mediated excision of the *floxneo* cassette. ES cell clones carrying the *R26^floxneoWnt4^* targeted allele were identified and chimeras were generated that transmitted the targeted allele to progeny. *R26^floxneoWnt4^* heterozygous and homozygous mutant mice appeared normal and were fertile.

**Figure 2:**
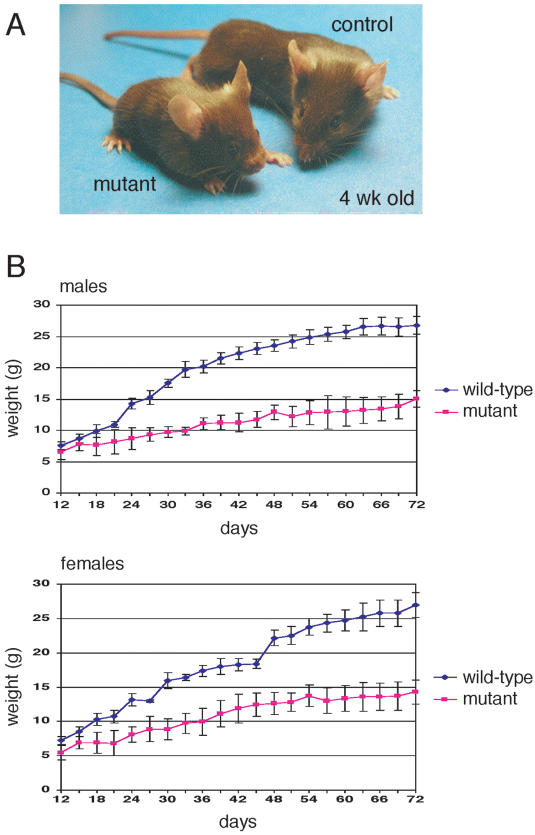
Growth defects in *R26^floxneoWnt4^; Col2a1-Cre* mutants. A, 4-week-old *R26^floxneoWnt4^; Col2a1-Cre* mutant and control littermates. The mutant has a significantly shorter body and altered head shape relative to the control. B, Mean body weight comparisons±standard error between sex-matched mice from 12 to 72 days after birth. n = 5 for mutants, and n = 6 for controls.

To activate the *Wnt4* transgene, *R26^floxneoWnt4^* heterozygotes were bred with *Col2a1-Cre* transgenic mice to generate *R26^floxneoWnt4^*; *Col2a1-Cre* double heterozygotes, hereafter designated mutants (**Fig. 1C**), that were obtained at the predicted Mendelian ratio (∼25%). The *Col2a1-Cre* transgene has been shown to initiate Cre reporter activity as early as 8.5 dpc [27]. All *R26^floxneoWnt4^*; *Col2a1-Cre* mutants were viable and developed a dwarf phenotype (**Fig. 2A**). Most of the mutants initiated growth defects beginning around 7 to 10 days after birth (data not shown). The body weights of mutants and controls were measured starting from postnatal day 12 (P12) to P72 at 3-day intervals (**Fig. 2B**). Male and female *R26^floxneoWnt4^*; *Col2a1-Cre* mutants had similar body weight growth rate characteristics. After weaning at 3 weeks of age, male mutants were approximately 50 to 60% and female mutants were approximately 60 to 70%, of the body weight of their age- and sex-matched controls (**Fig. 2B**).

**Figure 3:**
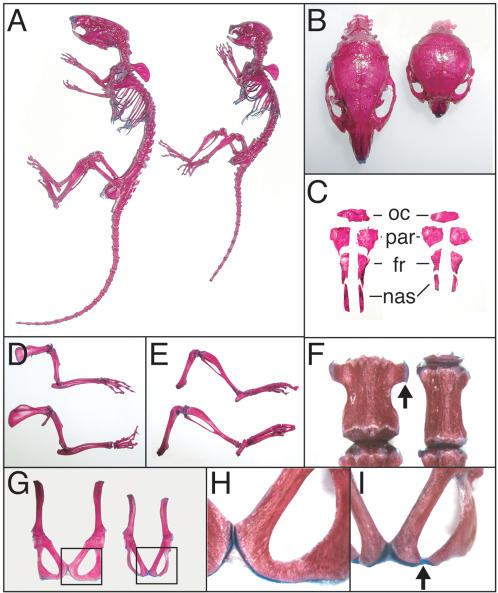
Skeletal defects in *R26^floxneoWnt4^; Col2a1-Cre* mutants. Skeleton preparations from 6-week-old *R26^floxneoWnt4^; Col2a1-Cre* mutants and *R26^floxneoWnt4^*controls. A, Intact skeletons, control (left) and mutant (right). B, Dorsal view of skulls, control (left) and mutant (right). C, individual dorsal skull bones, control (left) and mutant (right). D, Isolated forelimbs, mutant (top) and control (bottom). E, Isolated hindlimbs, mutant (top) and control (bottom). F, Third lumbar vertebrae, showing lateral ossifications (arrow) in the control (left) that are hypoplastic in the mutant (right). G, Isolated pelvic bones from 6-week-old mice, control (left) and mutant (right). H, Higher magnification of boxed region shown in panel G, showing pubic and ischial bone fusion of the control. I, Higher magnification of boxed region shown in panel G, showing lack of fusion between the pubic and ischial bones of the mutant. fr, frontal bone; nas, nasal bone; par, parietal bone; oc, occipital bone.

Skeleton preparations of 6-week-old mice were examined (**Fig. 3A**). In addition to shortened axial skeletons and limbs, *R26^floxneoWnt4^; Col2a1-Cre* mutants had smaller skulls (**Fig. 3B**). The skulls had a dome-shaped neurocranium vault, shorter viscerocranium, and a wider distance between the two orbits (**Fig. 3B**). Separation of the dorsal skull bones revealed the parietal bones to be fairly equal in size, the frontal and occipital bones to be slightly smaller, and the nasal bones to be significantly shortened in comparison to controls (**Fig. 3C**). The limbs of the *R26^floxneoWnt4^; Col2a1-Cre* mutants were disproportionately shorter than controls (**Fig. 3D, E**).

*R26^floxneoWnt4^; Col2a1-Cre* mutants also had lumbar vertebrae and pelvic bone defects. The vertebrae of 6-week-old mutants were narrow and flat as illustrated by the third lumbar vertebrae, which showed a reduction in lateral bone (**Fig. 3F**). The posterior region of the pelvic bone is composed of the pubic and ischial bones that are fused in 6-week-old controls (**Fig. 3G, H**). However, these bones retained cartilage between them in the mutants (**Fig. 3G, I**). Ossification of the cartilage between these two bones was present in 8-week-old mutants, although the pelvic bone was still thinner than controls.

**Figure 4:**
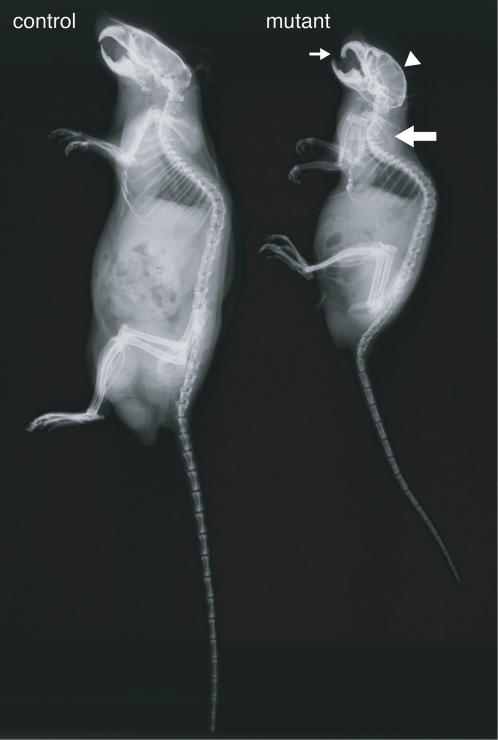
Radiographic analysis for 9-month-old *R26^floxneoWnt4^* *; Col2a1-Cre* mutants. The mutants had a smaller skeleton, abnormal skulls with a domed vault (arrowhead) and protruding incisors (small arrow). Kyphosis (large arrow) of the cervical-thoracic spine was also observed in the mutants. Control, *R26^floxneoWnt4^* heterozygote; mutant, *R26^floxneoWnt4^; Col2a1-Cre*.

Radiographic analyses of 9-month-old mice showed that the *R26^floxneoWnt4^; Col2a1-Cre* mutants had small skeletons, dome-shaped skulls, protruding incisors, and kyphosis of the cervical-thoracic spine (**Fig. 4**). In addition, the 9-month-old mutants moved slowly, suggesting that these skeletal abnormalities inhibited movement. No gross differences in bone mineralization were observed in X-ray images of mutants and controls (**Fig. 4**).

### Growth plate abnormalities in *R26^floxneoWnt4^; Col2a1-Cre* mutant mice

**Figure 5:**
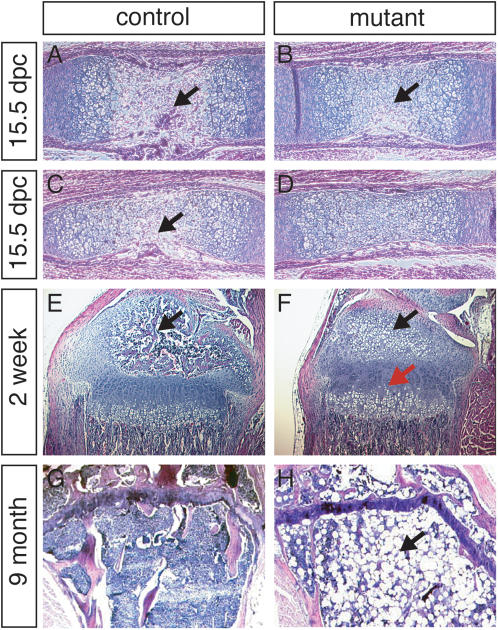
Histological analyses of long bones. A, C, E, G are H & E stained histological sections of *R26^floxneoWnt4^* heterozygous controls; and B, D, F, H are *R26^floxneoWnt4^; Col2a1-Cre* mutants. A, B, 15.5 dpc tibiae. C, D, 15.5 dpc femurs. The formation of the primary ossification center (arrows) was delayed in both tibiae and femurs of the mutant in comparison to controls. E, F, 2-week-old tibiae, showing development of the secondary ossification center (black arrow) in the control but only chondrocyte hypertrophy (black arrow) in the mutant that also had a disorganized growth plate (red arrow). G, H, 9-month-old mutant tibia with little bone marrow (G) filled with adipocytes (H, arrow).

Tibiae and femurs of 15.5 dpc mutant mice were examined by histology. At this stage of embryogenesis, the primary ossification centers (POC) have developed in both the tibiae and femurs of controls (**Fig. 5A, C**), with blood vessels originating from the perichondrium present in the metaphysis. However, at this stage of development in the mutants, the POC was not observed in tibiae and had just started to form in femurs (**Fig. 5B, D**). At stages later than 15.5 dpc, only sections of tibiae were used for comparisons. At P1 and P5, there were no apparent differences between mutants and controls (data not shown). Hypertrophic chondrocytes, ready to be invaded by blood vessels in the future secondary ossification center (SOC), were observed in controls at P7, but not in the mutants. At P10, the secondary ossification centers of the controls had started to form, and proliferating chondrocytes were organized in columns (data not shown). In the same area of the mutants, only hypertrophic chondrocytes were present. At P14, most mutants had initiated hypertrophic chondrocyte development in the SOC (**Fig. 5E, F**). The mutant growth plates were also less organized, with less columnar organization, and some hypertrophic chondrocytes had developed earlier than controls in the zone of proliferating chondrocytes (**Fig. 5E, F**). The timing of these histological alterations in bone formation correlate with the initial growth defects observed in the mutants.

At P14, there were distinct differences in the chondrocyte zones between *R26^floxneoWnt4^; Col2a1-Cre* mutants and controls. The proliferating chondrocyte zone in the tibiae of controls were larger than mutants, yet the hypertrophic chondrocyte zone in tibiae of mutants were larger than controls. At 3 weeks of age, both mutants and controls have developed SOCs in tibiae, though they were better developed in controls than in the mutants (data not shown). At 9 months of age, the tibiae of mutants (n = 2) were deficient in bone marrow and were filled with adipocytes in epiphyseal and metaphyseal regions (**Fig. 5G, H**). In contrast, inspection of 12-month-old control mice (n = 2) showed metaphyseal regions full of bone marrow (data not shown).

**Figure 6:**
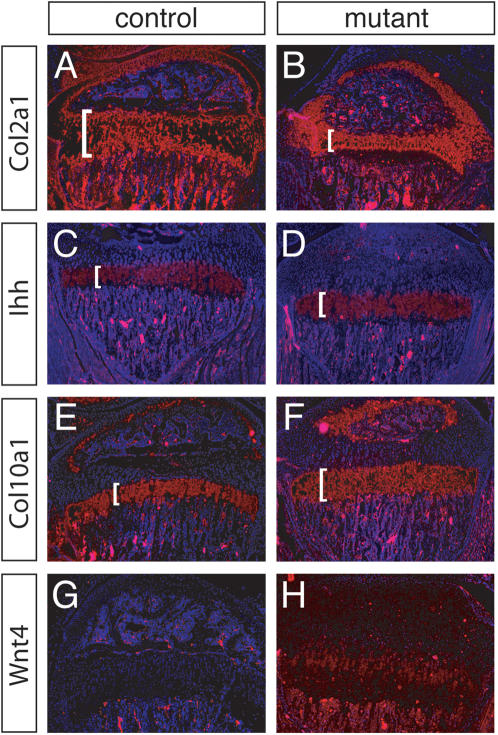
Section RNA in situ hybridization of tibial growth plates of 3-week-old animals. Molecular marker analysis of tibiae of 3-week-old *R26^floxneoWnt4^* heterozygous control (A, C, E, G) and *R26^floxneoWnt4^; Col2a1-Cre* mutant (B, D, F, H) mice. *Col2a1* marks proliferating and prehypertrophic chondrocytes; *Col10a1* marks prehypertropic chondrocytes; *Ihh* marks prehypertropic and hypertropic chondrocytes. Brackets mark relevant regions. *Wnt4* hybridization is undetectable in the growth plate of the control, whereas *Wnt4* transcripts are detected throughout the growth plate of the mutant.

We employed section in situ hybridization using several molecular markers, to study the chondrocyte zones in 3-week-old tibiae. *Col2a1* is expressed in proliferating and prehypertrophic chondrocytes. *Col2a1* transcripts were detected in a smaller region in the mutants relative to controls, indicating that tibiae of 3-week-old mutants have a narrower zone of proliferating and prehypertrophic chondrocytes (**Fig. 6A, B**). *Indian hedgehog (Ihh)*, a member of the *Hedgehog* gene family, is a key molecule in endochondral ossification [29]. At postnatal stages, *Ihh* is expressed predominantly in prehypertrophic chondrocytes. Hybridization of *Ihh* showed no obvious differences in mutant tibiae relative to controls (**Fig. 6C, D**), suggesting that the narrower zone defined by *Col2a1* expression is predominantly due to a reduced proliferating chondrocyte zone. *Col10a1* is a marker of prehypertrophic and hypertrophic chondrocytes, cells that have exited the cell cycle [29]. Hypertrophic chondrocytes form the terminal zone of the growth plate that is poised to become apoptotic and replaced by bone. *Col10a1* hybridized to a significantly larger zone in the mutant growth plates in comparison to controls, indicating a larger proportion of hypertrophic chondrocytes relative to controls (**Fig. 6E, F**).

Gene expression of *Wnt4* during skeletal development has been described previously. In chick, *Wnt4* expression is first detected at embryonic stages in the joint regions between two long bones [5]. Another study showed *Wnt4* expression in hypertrophic chondrocytes at later stages [18]. Using high-stringency for in situ hybridization, *Wnt4* transcripts were not detected in the growth plates of 3-week-old control mice, but were found in almost the entire cell population of the growth plates of the mutants (**Fig. 6G, H**). Although we have not determined the earliest stage that the *Wnt4* transgene is activated by the *Col2a1-Cre* transgene these results suggest that Cre acts in chondrogenic precursors to activate *Wnt4* transgene expression in all cells of the growth plate.

**Figure 7:**
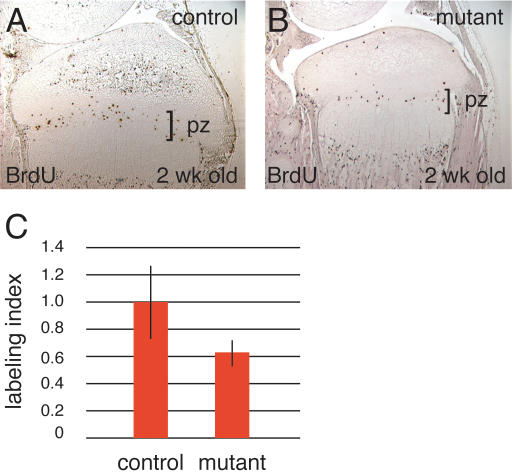
Cell proliferation in the epiphyseal growth plate of the proximal tibia. A, B, BrdU-labeled cells in 2-week-old control and *R26^floxneoWnt4^; Col2a1-Cre* mutant mice. C, The fraction of chondrocytes in the proliferation zone (pz) that incorporated BrdU was 0.189±0.051 in control mice compared to 0.122±0.020 in mutants (P<0.001). The labeling percentage of the control was designated as 1.0, and the relative percentage of the mutant was calculated as 0.64.

Chondrocyte proliferation was examined in 2-week-old mutants and controls by BrdU-labeling (**Fig. 7A, B**). BrdU-labeling revealed that the fraction of chondrocytes in the zone of proliferation that incorporated BrdU was 0.189±0.051 in controls but only 0.122±0.002 in mutants, resulting in a mitotic index of 0.64 for the *R26^floxneoWnt4^; Col2a1-Cre* mutants (**Fig. 7C**). This indicates that overexpression of *Wnt4* leads to decreased chondrocyte proliferation during tibial growth.

### Decreased VEGF expression in *R26^floxneoWnt4^; Col2a1-Cre* mutant mice

**Figure 8:**
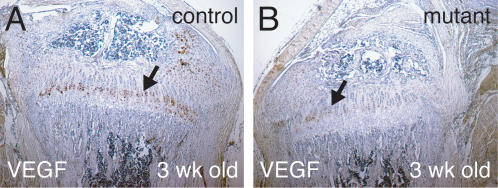
VEGF immunohistochemistry of 3-week-old tibiae. A, B, VEGF immunostaining (brown) in growth plates of 3-week-old tibiae. A, The control stained strongly for VEGF in prehypertrophic and hypertrophic chondrocytes (arrow). B, VEGF immunostaining in the *R26^floxneoWnt4^; Col2a1-Cre* mutant tibia was weaker and restricted to prehypertrophic chondrocytes (arrow).

During endochondral bone formation, VEGF induces angiogenesis from the perichondrium. In mouse, VEGF has been reported to be secreted by hypertrophic chondrocytes [30]. However, VEGF immunostaining in 3-week-old wild-type tibiae was not restricted to the terminal hypertrophic chondrocytes, but rather was predominantly expressed in prehypertrophic and early hypertrophic chondrocytes (**Fig. 8A**). In *R26^floxneoWnt4^; Col2a1-Cre* mutants, VEGF immunostaining was weak in prehypertrophic chondrocytes and almost absent in hypertrophic chondrocytes (**Fig. 8B**).

## Discussion

### An in vivo system to tissue-specifically overexpress *Wnt4* in mice

*Wnt4* was initially shown to be essential for kidney tubulogenesis for nephron formation [16] and differentiation of the female gonad and reproductive tract [17]. Subsequently in chick, *Wnt4* expression in the joint regions and misexpression studies indicated a role in skeleton development [5]. To further study *Wnt4* function during development, we used a Cre/*lox*P system to conditionally express *Wnt4* from the ubiquitously-expressed *Rosa26* locus potentially in any mouse tissue in a Cre-dependent manner. To study the activity of *Wnt4* in skeletal development, we used *Col2a1-Cre* transgenic mice that express Cre in chondrogenic tissues [27]. Examination of *Wnt4* expression in the *R26^floxneoWnt4^; Col2a1-Cre* mutants suggested that Cre activates *Wnt4* transcription in chondrogenic precursors, leading to *Wnt4* transgene expression throughout the entire growth plate. These findings suggest that the *R26^floxneoWnt4^* mice may also be useful for *Wnt4* misexpression studies in other tissues.

### Overexpression of *Wnt4* alters the growth plate

The external morphologies of the *R26^floxneoWnt4^; Col2a1-Cre* mutants were similar to mice with mutations in *Fgfr3*, *Mt1-Mmp*, and *Link protein* (*Hapln1*) [31]–[36]. The length of the growth plates of the *R26^floxneoWnt4^; Col2a1-Cre* mutants was nearly identical to wild type, although with an altered appearance. *R26^floxneoWnt4^; Col2a1-Cre* mutants have an expanded zone of hypertrophic chondrocytes and a smaller zone of proliferating chondrocytes. Overexpression of *Wnt4* also causes a decrease in VEGF expression that may result in a reduction of vascularization that in turn leads to delayed formation of primary and secondary ossification centers. The *Col2a1-Cre* transgene is active in chondrocyte precursors of endochondral bones [27]. Interestingly, the skulls of the *R26^floxneoWnt4^; Col2a1-Cre* mutants were significantly smaller than controls. The skull is composed of elements derived from both endochondral and membranous bone formation. The alterations observed in the membranous bones of the mutant skulls may be indirect effects of altered endochondral skull bones. All of the skeletal alterations mentioned above likely contribute to the development of the dwarf phenotype.

*Wnt4* is expressed in the developing joint regions and a subset of hypertrophic chondrocytes [5], [18], [19]. *Wnt4* homozygous mutant mice die within 24 hours after birth due to severe defects in kidney function [16], but no skeletal abnormalities have been reported [9]. However, *Wnt4*/*Wnt9a* double mutants show some joint cell identity abnormalities [9], [10]. The perinatal lethality precludes studies of the role of *Wnt4* in the skeleton after birth. Such studies will require the generation of a *Wnt4* conditional null allele [37]. Retroviral-mediated *Wnt4* misexpression in chick limbs accelerated chondrocyte maturation; in contrast, *Wnt5a* misexpression in the same model inhibited chondrocyte maturation [5]. Viral-mediated misexpression of β-catenin or a constitutive-active form of LEF/TCF display phenotypes similar to *Wnt4* misexpression, suggesting that *Wnt4* influence on limb shortening may be mediated by β-catenin [13]. In addition, viral-mediated misexpression of the Wnt-antagonist Frzb-1 in growth plates delayed chondrocyte differentiation [12]. Together these data imply that endogenous *Wnt4* signaling may have a role in chondrocyte maturation.

We show that overexpression of *Wnt4* in the mouse growth plate may also influence chondrocyte maturation, as shown by reduced zones of proliferating chondrocytes and expanded zones of *Col10a1* hybridization and hypertrophic chondrocytes in *R26^floxneoWnt4^; Col2a1-Cre* mutants. In addition, in growth plates of *R26^floxneoWnt4^; Col2a1-Cre* mutants, the zone of *Col2a1* hybridization was significantly smaller yet the *Ihh* zone essentially unchanged, indicating a decrease in proliferating chondrocytes. The decrease of the zone of proliferating chondrocytes may result from lower rate of proliferation in mutants or a higher percentage of cells exiting the proliferation state. However, an in vitro micromass culture assay has shown that infection by a retroviral-delivered *Wnt4* did not decrease the rate of cell proliferation [18]. In addition, no detectable change of cell proliferation was observed by *Wnt5a* and *5b* misexpresssion using the same system. Indeed, recent studies in transgenic mice demonstrated that *Wnt5b* could promote proliferation of chondrocytes in vivo [11]. These distinct results may reflect the difference between mouse and chick models, or differences between the in vitro and in vivo approaches. The ability of *Wnt4* to decrease the zone of proliferating chondrocytes may represent an enhancement of the endogenous activity of *Wnt4*, a competition with the activity of *Wnt5b* that promotes proliferation of chondrocytes, or a mimicking of *Wnt5a* function that inhibits the transition from resting chondrocytes to proliferating chondrocytes. Moreover, since *Wnt4* may accelerate the differentiation of chondrocytes, proliferating chondrocytes with overexpression of *Wnt4* may exit the cell cycle rapidly, leading to narrower zone of proliferating chondrocytes.

The *R26^floxneoWnt4^; Col2a1-Cre* mutants also had disorganized growth plates. The organized structure of the growth plate is tightly linked between the chondrocytes and the extracellular matrix (ECM) and Wnt actions on cell adhesion have been proposed [38]. Overexpression of *Wnt4* may interrupt such a relationship, by either changing the cell membrane structure of chondrocytes or altering components of the ECM. *Wnt4* has been shown to signal canonically, non-canonically, or through neither pathway, depending upon the experimental context [39]–[44]. It is not clear which pathway(s) is utilized for the *Wnt4*-induced dwarfism documented in our study.

### Overexpression of *Wnt4* and skeleton vascularization

The growth plates of *R26^floxneoWnt4^; Col2a1-Cre* mutants displayed decreased VEGF expression. VEGF is a key regulator for vascularization and plays an important role during endochondral bone formation, where VEGF couples hypertrophic cartilage remodeling, ossification, and angiogenesis [30]. *Vegf* heterozygous mice die at early embryonic stages [45], [46], but animals that express only the VEGF^120^ isoform can survive to term. *Vegf* ^120/120^ mutants appeared to have low angiogenesis activity [47], [48], and share similar phenotypes with *R26^floxneoWnt4^; Col2a1-Cre* mutants. These abnormalities include delayed invasion of vessels into the primary and secondary ossification centers, reduction in mineralization of mutant bone, and expansion of the zone of hypertrophic chondrocytes. Thus, these phenotypes observed in the *R26^floxneoWnt4^; Col2a1-Cre* mutants may be caused by overexpression of *Wnt4*, causing reduced expression of VEGF.

Although the relationship between Wnt proteins and VEGF during skeletal development has not yet been clarified, Wnt/β-catenin signaling has been suggested to play a role in activation of VEGF gene expression in benign colonic adenomas in which mutant activated Wnt/β-catenin pathway is often associated with up-regulated VEGF [49]. Moreover, after transfection of a dominant-negative form of TCF4, one of the downstream molecular components of Wnt signaling, VEGF expression was repressed. Truncated VE-cadherin in mice, lacking the β-catenin-binding cytosolic domain, impaired VEGF-mediated angiogenesis [50]. These results indicated that Wnt signaling mediated by the β-catenin pathway could activate VEGF function, but this in contradiction with the *R26^floxneoWnt4^; Col2a1-Cre* mutant phenotype. However, *Wnt* gene family members often exert distinct functions in a particular tissue of the same stage. For instance, infection of chick limbs using a retrovirus carrying *Wnt5a* or *Wnt4* presented distinct effects, with *Wnt5a* delaying chondrocyte differentiation, whereas *Wnt4* accelerated it [5]. Also, *Wnt5b* promoted the transition of resting chondrocytes to proliferating chondrocytes, whereas *Wnt5a* inhibited it [11]. This may reflect unique activities of *Wnt* genes, or endogenously, Wnt proteins may function as antagonists of each other.

*Wnt4* is an important regulator of female reproductive organ development in mice. The ovaries of female mice lacking *Wnt4* were masculinized with indications of Leydig cell differentiation [17]. In addition, the mutant ovaries had a large coelomic blood vessel, a primary characteristic of the testis [51]. This has led to the proposal that *Wnt4* may repress angiogenesis in developing female gonads, blocking the testis differentiation pathway.

The dwarfism of *R26^floxneoWnt4^; Col2a1-Cre* mutants became apparent only after birth. However, skeletal changes were found earlier in development, consistent with the activity of the *Col2a1-Cre* transgene at the initial stages of cartilage formation [27]. The apparently weak response to transgenic *Wnt4* at fetal stages may be attributable to limited expression of Wnt4 receptors or an excess of Wnt inhibitors. However, the overt dwarfism of the *R26^floxneoWnt4^; Col2a1-Cre* mutants may be the result of VEGF insufficiency. Neonatal mice homozygous for a *Vegf* 120 isoform allele had 10% shorter tibiae, and slight differences in bone length were detected at 16.5 dpc in comparison to controls [47]. *R26^floxneoWnt4^; Col2a1-Cre* mutants may have stronger VEGF activity than *VEGF^120/120^* mutants because the phenotypes displayed in *VEGF^120/120^* mutants were more severe than those of *RWnt4; Col2a1-Cre* mutants. For example, the expansion of the zone of hypertrophic chondrocytes was larger, and the delayed time of formation for the primary ossification center was longer in *Vegf^120/120^* mutants. Thus, it may be reasonable to expect that the shortening of tibial length observed in *R26^floxneoWnt4^; Col2a1-Cre* mutants as in *Vegf* ^120/120^ mutants becomes apparent only after birth.

In summary, *Wnt4* is expressed during skeleton development. The studies presented here demonstrate that dysregulated *Wnt4* expression in chondrogenic tissues leads to skeletal defects and dwarfism in mice. The data indicate that *Wnt4* levels must be regulated in chondrocytes for normal growth plate development and skeletogenesis. In addition, these studies suggest that pathologies that lead to Wnt overexpression may influence chondrogenic tissues.

## Materials and Methods

### Generation of *R26^floxneoWnt4^* mice

The mouse *Wnt4* cDNA encoding the entire open reading frame was isolated by RT-PCR. RNA from 13.5 dpc gonads from mouse strain 129/EvSvTac was isolated and used to synthesize cDNA. The *Wnt4* cDNA was subsequently amplified by two rounds of PCR. The first primer set was: forward 5′-CCGCGCGGCGAAAACCTG-3′ and reverse 5′-CTGTTTAAGTTATTGGCCTTC-3′. The second primer set was: forward 5′-GCCTTGGGATCCCTGCCCCGGGCTGG-3′ and reverse 5′-ACGCAGGCGGCCGCACTAGTCCTAGGCATGGTCA-3′. The final PCR product was subcloned into the *Bam*HI and *Not*I sites of pBluescript KS(-) and sequenced.

The pR26-1 plasmid [22] was used to insert a conditional *Wnt4* expression cassette into the *Rosa26* locus. The expression cassette begins with a splice acceptor sequence (*SA*), followed by *Pgkneo* and five polyadenylation sequences flanked by *loxP* sites (floxneo). The mouse *Wnt4* cDNA followed by a bovine growth hormone polyadenylation (*bpA*) sequence was placed 3′ of floxneo. The expression cassette, *SA-loxP-Pgkneo-5pA-loxP-Wnt4-bpA,* was inserted into the *Xba*I site of pR26-1, to generate the gene targeting vector (**Fig. 1A**). A diphtheria toxin expression cassette (*DT*) is present within pR26-1 for negative selection. The targeting vector was linearized with *Kpn*I and electroporated into AB1 ES cells and selected in G418 [52]. ES cell clone genomic DNAs were digested with *Xba*I and analyzed by Southern blot [53] using a 5′ external probe as described [22] and a 3′ internal probe using *bpA* (**Fig. 1B**). Targeted ES cell clones were injected into C57BL/6 (B6) blastocysts to generate chimeras that transmitted the *R26^floxneoWnt4^* allele to their progeny. The *R26^floxneoWnt4^* allele was examined on a B6129 mixed genetic background. The *Col2a1-Cre* transgenic mice were originally generated on a B6SJLF2 genetic background but have been backcrossed to B6 for >5 generations. All procedures using animals were approved by the Institutional Animal Care and Use Committee.

### Mouse genotyping

The *R26^floxneoWnt4^* allele and *Col2a1-Cre* transgene were genotyped by PCR using tail DNA (**Fig. 1C**). Two primer sets were used in a single PCR reaction to identify the *R26^floxneoWnt4^* allele and the *Col2a1-Cre* transgene. Primers for identifying the *R26^floxneoWnt4^* allele were, *R26-R1*: 5′-AAAGTCGCTCTGAGTTGTTAT-3′, *R26-R2*: 5′-GCGAAGCGTTTGTCCTCAACC-3′, and *R26-R3*: 5′-GGAGCGGGAGAAATGGATATG-3′. Primers for identifying the *Col2a1-Cre* transgene were, 5′- TCCAATTTACTGACCGTACACCAA-3′, and 5′- CCTGATCCTGGCAATTTCGGCTA-3′. PCR amplification was 40 cycles of 94°C for 30 sec, 62°C for 5 sec and 68°C for 60 sec. Approximately 600-bp and 350-bp fragments were amplified for the wild-type *Rosa26* and targeted alleles, respectively. The *Col2a1-Cre* transgene was identified as a ∼550-bp fragment.

### Skeleton preparations and histology

6-week-old mice were prepared to visualize bone by alizarin red staining as described [54]. For histological analysis, tissues were fixed in 4% paraformaldehyde at 4°C overnight for pups at postnatal day 7 (P7) or younger or for two days for pups older than P7. After fixation, samples were washed twice in PBS and decalcified with 0.5 M EDTA, pH 8.0 for 7 to 14 days at 4°C prior to dehydration and paraffin embedding. 7 mm sections were cut and stained with hematoxylin and eosin (H&E).

### Radiographic analysis

4 and 9 month-old mice were sacrificed by CO_2_ asphyxiation. Skeleton morphology was imaged at 27 KV for 20 sec using a Cabinet X-Ray System (Faxitron X-ray Corporation, Wheeling, Illinois).

### Immunohistochemistry

Skeletal tissues were treated with hyaluronidase (0.4% in PBS, pH 5.0) to unmask epitopes for immunohistochemistry. Immunostaining was performed using the Vectastain ABC kit (Vector Labs, Burlingame, California), according to the manufacturer's instructions. Rabbit anti-mouse VEGF antibody (Santa Cruz Biotechnology, Santa Cruz, California) was diluted 1∶20.

### Cell proliferation

Mice were injected intraperitoneally with bromodeoxyuridine (BrdU) at 100 mg/g body weight and sacrificed 1 hour after injection. Histological sections of skeletal tissues were prepared and immunostained for BrdU (Oncogene Research Products, San Diego, CA). The proliferation rate was calculated as the number of BrdU-labeled cells divided by the total number of cells in the same microscopic field.

### RNA in situ hybridization of histological sections

The procedures for RNA in situ hybridization of histological sections were adapted from those described [55]. ^35^[S]-UTP-labeled antisense or sense RNA probes were prepared. After hybiridzation, samples were exposed for 7 to 40 days and then developed using Kodak D-19 developer and fixer, and counterstained with Hoechst dye.
